# Acute transverse myelitis and psoriasiform dermatitis associated with Sjoegren’s syndrome: a case report

**DOI:** 10.1186/1756-0500-7-580

**Published:** 2014-08-29

**Authors:** Carolin Kurz, Silke Wunderlich, Derek Spieler, Benedikt J Schwaiger, Christian Andres, Claudia Traidl-Hoffmann, Rüdiger Ilg

**Affiliations:** Department of Neurology, Klinikum rechts der Isar, Technische Universität, Ismaninger Strasse 22, Munich, 81675 Germany; Helmholtz Zentrum München, Institute of Human Genetics, Ingolstädter Landstrasse 1, Neuherberg, 85764 Germany; Department of Neuroradiology, Klinikum Rechts der Isar, Technische Universität München, Ismaninger Strasse 22, Munich, 81675 Germany; Department of Dermatology, Klinikum Rechts der Isar, Technische Universität München, Biedersteiner Strasse 29, Munich, 80802 Germany; Institute of environmental medicine, UNIKA-T, Technische Universität München, Munich, 80802 Germany

**Keywords:** Sjögren’s syndrome, Sjoegren’s syndrome, Myelitis, Psoriasis, Psoriasiform dermatitis, Cyclophosphamide, Rituximab

## Abstract

**Background:**

Clinical complications of Sjoegren’s syndrome include myelitis and skin manifestations. There is scarce observational data and a lack of randomised controlled studies regarding the treatment of Sjoegren’s syndrome in the presence of such complications.

**Case presentation:**

Here we report the case of a 41-year-old Caucasian female patient with biopsy-proven Sjoegren’s syndrome who initially presented with generalized exanthema and subsequently developed acute extensive transverse myelitis. In view of the rapid deterioration we opted for an intensive treatment using a combination of corticosteroid pulse therapy, plasmapheresis and cyclophosphamide, which we later changed to rituximab. Under that treatment the skin manifestations resolved entirely whereas transverse myelitis showed incomplete remission.

**Conclusion:**

Severe neurological and dermatological complications may occur in Sjoegren’s syndrome. This suggests a close yet currently unclear pathogenetic relationship. Intensive immunosuppressant treatment resulted in significant improvement of both symptom clusters. Skin manifestations may precede other severe complications in Sjoegren’s syndrome and therefore require particular attention.

## Background

Sjoegren’s syndrome (SS) is an autoimmune disease that primarily affects the exocrine glands and leads to keratoconjunctivitis and xerostomia [1]. Systemic manifestations may also occur including myoarthralgia, vasculitis and dermatological findings such as dry skin, urticaria or cutaneous vasculitis [1–5]. SS affects the nervous system in approximately 20% of cases and rarely causes severe complications like acute transverse myelitis [6].

The co-existence of SS and psoriasis has rarely been described before, the mutual immunological factors being unclear [7]. The management of SS with neurological or dermatological complications is still a matter of debate. However, it is known that cases with myelitis require particularly intensive treatment [1–3, 5, 8]. In such cases cyclophosphamide appears to be most effective for achieving symptom remission [1–3, 5]. In addition, limited experience suggests that patients with extraglandular manifestations of SS benefit from long-term treatment with rituximab [8, 9].

Here we report the case of a 41-year-old female patient with biopsy-proven SS who rapidly developed severe exanthema and extensive transverse myelitis.

## Case presentation

**Figure 1:**
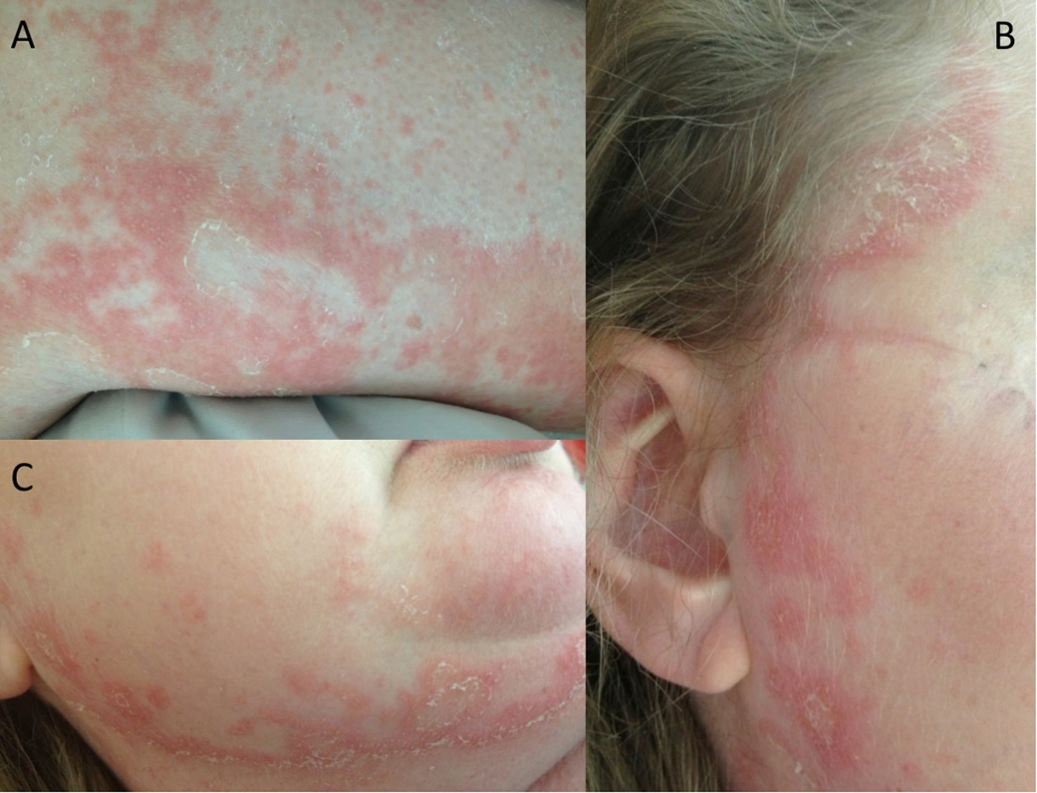
**Macroscopic dermatological findings: maculopustular and squamous exanthema of the entire integument. (A)** Left leg **(B)** cheek and forehead **(C)** chin.

A 41-year-old Caucasian woman was admitted to the department of dermatology with generalized maculopapular exanthema (Figure 1). Within three days she developed fatigue, headache, incomplete third cranial nerve palsy, urinary retention, paraparesis (strength 1-2/5 bilaterally) and sensory loss below level T4. Subsequently, tetraparesis (strength arms 3/5, legs 1/5) and dyspnoea with reduced lung capacity emerged. In her past medical history SS had been suspected since the patient had suffered from xerophthalmia, xerostomia and myoarthralgias since the late 1990’s and SS-A-antibodies had been tested positive. The patient had temporarily been treated with oral corticosteroids for sicca symptoms two years before admission.

### Diagnostic findings

**Figure 2:**
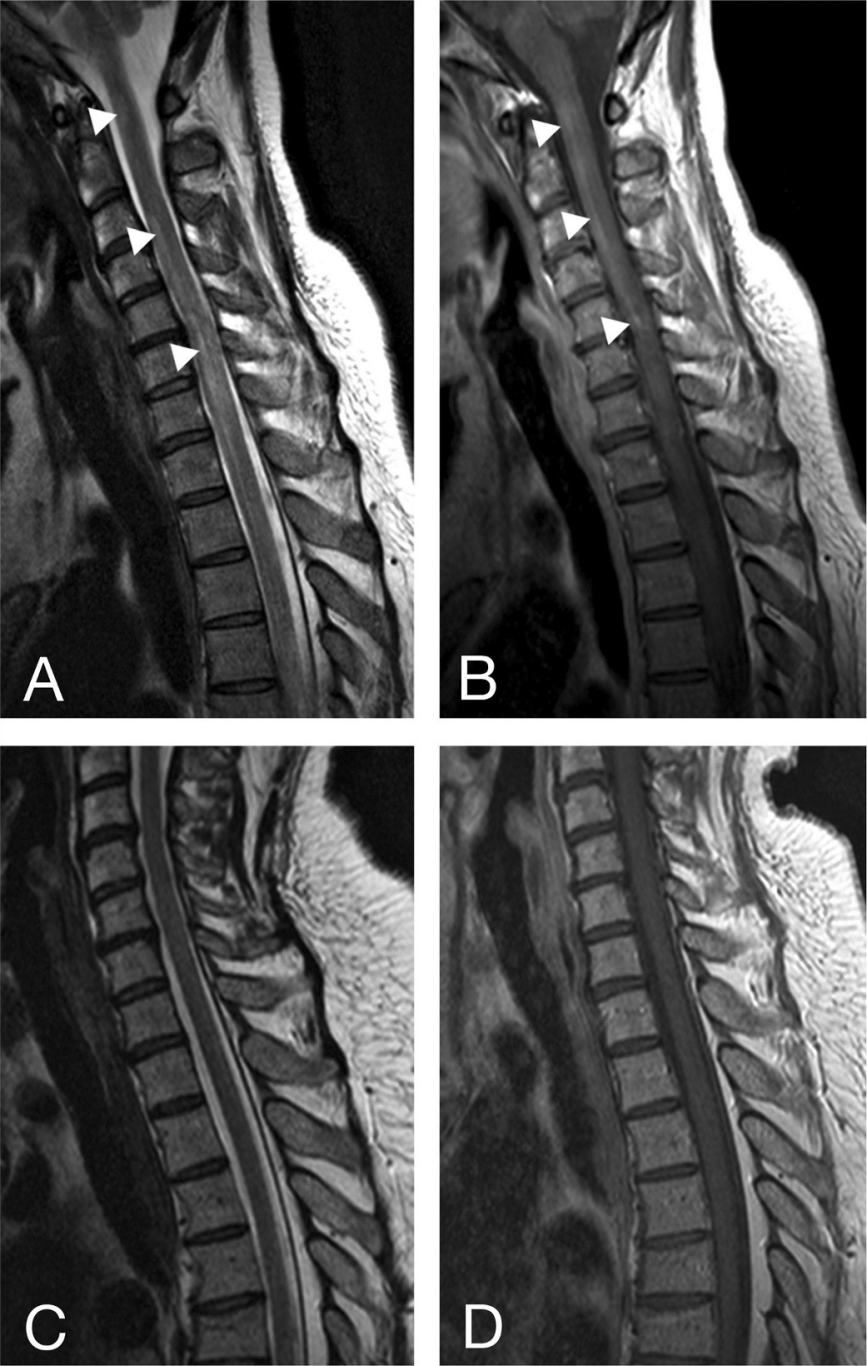
**Sagittal T2-weighted (A) and T1-weighted, gadolinium enhanced (B) MRI pictures: Confluent contrast-enhanced lesions in the cervical spinal cord extending to the caudal medulla oblongata (A and B, white arrows); no evidence of myelitis after cyclophosphamide therapy (C and D).**

**Figure 3:**
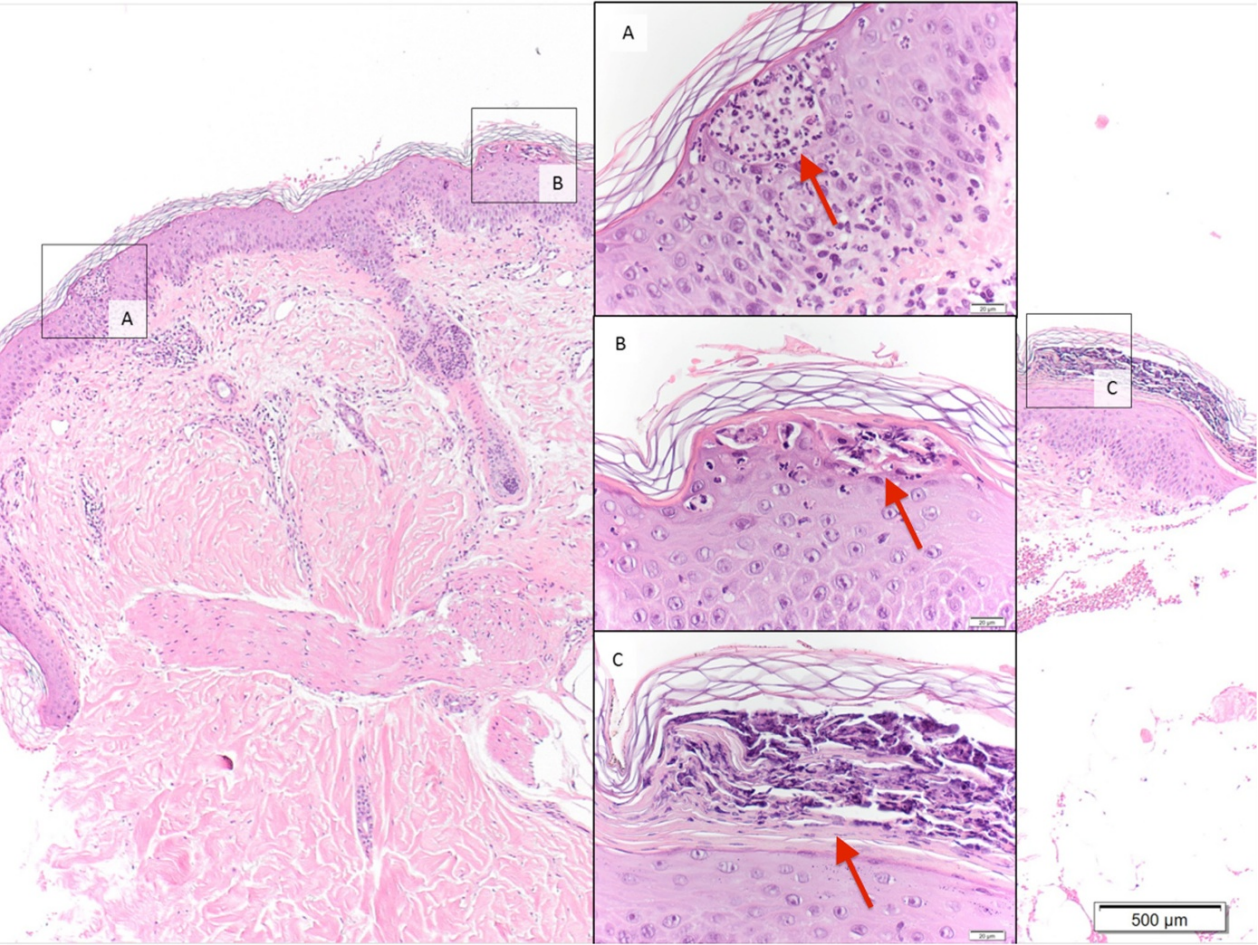
**Histological findings (hematoxylin-eosin staining; overview 40x, inset 200x; Punch biopsy from left upper arm): neutrophils and lymphocytes forming intraepithelial pustules (inset A, red arrow), aggregated neutrophilic granulocytes in parakeratotic foci (inset B, red arrow), orthokeratosis associated with parakeratosis and mild superficial perivascular lymphocytic infiltrates around dilated vessels (insert C, red arrow).**

Magnetic resonance (MR) imaging revealed extensive transverse myelitis reaching from the caudal medulla oblongata to level C7 (Figure 2A and B) as the cause of tetraparesis. Cerebrospinal fluid (CSF) showed mild lymphomonocytic pleocytosis and impairment of the brain-blood-barrier. CSF culture was negative as were all polymerase chain reaction studies of the CSF for fungal and viral infection. Furthermore cytology of CSF was negative for malignancy. Identical oligoclonal bands in serum and CSF indicated systemic inflammation. Testing for human immunodeficiency virus (HIV) was negative. Anti-nuclear antibodies were elevated (1: 960) but all other tests for autoimmune antibodies were negative including aquaporin 4. The previous finding of positive SS-A antibodies was not replicated in our laboratory. This discrepancy is unusual but can be explained by different test sensitivities. The diagnosis of SS was confirmed according to consensus criteria by labial salivary gland biopsy, positive Schirmer’s test and salivatory gland scintigraphy [10]. Erythematous macules and papulosquamous lesions characterized the clinical appearance of the skin lesions. For further examination, a skin biopsy was performed and showed psoriasiform dermatitis with aggregated neurophilic granulocytes in parakeratotic foci (Figure 3, insets B and C) and exocytosis of neutrophils presenting as an intraepithelial pustule (Figure 3, inset A).

### Differential diagnosis

Concerning the neurological differential diagnosis, there was no history of previous radiation to the spine or clinically apparent optic neuritis, aquaporin 4 antibodies were negative and there were no brain abnormalities suggestive of MS on magnetic resonance imaging (MRI). Furthermore, we could neither find evidence for an arterial occlusion, a compressive aetiology or a viral or fungal infection nor another connective tissue disease -especially leukocytoclastic vasculitis or lupus erythematosus. Subacute cutaneous lupus erythematosus (SCLE) as an important differential diagnosis of psoriasiform dermatitis was excluded since histological characteristics for SCLE (liquefactive degeneration of the basal layer, edema of the upper dermis, scattered interface, perivascular and periadnexal lymphocytic infiltrates) were absent. In synopsis of all clinical and serological findings we considered the extensive transverse myelitis to be SS-associated.

### Treatment and outcome

Due to the rapid worsening of the patient’s condition and the evidence of severe demyelination on MRI we initiated seven sessions of plasmapheresis and simultaneous corticosteroid pulse therapy (1 gr/d for 5 days followed by oral tapering). With this treatment regimen tetraparesis and dyspnoea gradually improved but generalized exanthema worsened. For long-term therapy we chose cyclophosphamide (8 cycles). When reaching the maximum cumulated dose of cyclophosphamide after 6 months we switched to rituximab (375 mg/m^2^ every six months). Since then, the patient is regularly seen for the administration of rituximab and has had no relapse so far. The combination of plasmapheresis, corticosteroid pulse therapy and subsequent immunosuppression significantly improved the severe symptoms, but only an incomplete remission was achieved: while moderate paraparesis with decreased sensation of both legs and urinary retention remained (strength 3/5 bilaterally), generalized maculo-papulosquamous exanthema, dyspnea and oculomotor impairment resolved completely. A follow-up MRI after cyclophosphamide therapy showed significant improvement and no evidence of myelitis (Figure 2C and D).

## Conclusion

We report a patient with biopsy-proven SS who presented with an acute rash, acute transverse myelopathy and coincident cranial neuropathy. It is likely that the psoriasiform dermatitis represents a skin manifestation of SS. Hence, fulminant skin manifestations in SS may herald severe organ involvement and therefore require particular attention. Cutaneous manifestations of SS described to far are dry skin, immunological inflammatory conditions such as vasculitis and hypergammaglobulinaemic purpura. In rare cases, SS is associated with neutrophilic, granulomatous disorders. The pathogenetic relationship between neurological and dermatological manifestations remains elusive and needs further investigation. In particular, the role of circulating Th17 cells, which are known to play a major role in both Sjoegren’s syndrome and psoriasis needs to be clarified [11–13]. Following this, anti-interleukin-17 (anti-IL17) treatment could be an option for therapy. Of note recent findings demonstrate a T cell epitope mimicry between Sjögren’s syndrome Antigen A (SSA)/Ro60 and skin bacteria [14].

Due to the lack of randomised-controlled studies, the optimal treatment of SS cases with central nervous system (CNS) involvement remains unclear. In our case, high dose corticosteroids, plasmapheresis and long-term immunosuppression with cyclophosphamide followed by rituximab was obviously effective in stopping the suspected autoimmune inflammation but could not reverse the neurological damage. Therefore, in any case of unclear myelopathy, the coincidence of skin manifestations and cranial neuropathies should serve as a red-flag for an autoimmune disorder and should result in further screening for autoimmune disorders including SS.

## Consent

Written informed consent was obtained from the patient for publication of this Case Report and any accompanying images. A copy of the written consent is available for review by the Editor-in-Chief of this journal.

## Abbreviations

anti-IL17: Anti-interleukin-17
CNS: Central nervous system
CSF: Cerebrospinal fluid
HIV: Human immunodeficiency virus
MRI: Magnet resonance imaging
SCLE: Subacute cutaneous lupus erythematosus
SS: Sjoegren’s syndrome.
